# Contrasting Eutrophication Risks and Countermeasures in Different Water Bodies: Assessments to Support Targeted Watershed Management

**DOI:** 10.3390/ijerph14070695

**Published:** 2017-06-29

**Authors:** Tong Li, Chunli Chu, Yinan Zhang, Meiting Ju, Yuqiu Wang

**Affiliations:** College of Environmental Science and Engineering, Nankai University, Tianjin 300350, China; love777_happy@126.com (T.L.); zhangyn2011@whu.edu.cn (Y.Z.); jumeit@nankai.edu.cn (M.J.); yqwang@nankai.edu.cn (Y.W.)

**Keywords:** eutrophication, single factor evaluation, comprehensive eutrophication state index, watershed management

## Abstract

Eutrophication is a major problem in China. To combat this issue, the country needs to establish water quality targets, monitoring systems, and intelligent watershed management. This study explores a new watershed management method. Water quality is first assessed using a single factor index method. Then, changes in total nitrogen/total phosphorus (TN/TP) are analyzed to determine the limiting factor. Next, the study compares the eutrophication status of two water function districts, using a comprehensive nutritional state index method and geographic information system (GIS) visualization. Finally, nutrient sources are qualitatively analyzed. Two functional water areas in Tianjin, China were selected and analyzed: Qilihai National Wetland Nature Reserve and Yuqiao Reservoir. The reservoir is a drinking water source. Results indicate that total nitrogen (TN) and total phosphorus (TP) pollution are the main factors driving eutrophication in the Qilihai Wetland and Yuqiao Reservoir. Phosphorus was the limiting factor in the Yuqiao Reservoir; nitrogen was the limiting factor in the Qilihai Wetland. Pollution in Qilihai Wetland is more serious than in Yuqiao Reservoir. The study found that external sources are the main source of pollution. These two functional water areas are vital for Tianjin; as such, the study proposes targeted management measures.

## 1. Introduction

Eutrophication is a significant worldwide environmental problem. Eutrophication is a form of water pollution, caused by excessive loading of dissolved and particulate organic matter and inorganic nutrients (C, N, and P). Eutrophication risk refers to the possibilities and consequences of eutrophication [1]. Visible effects of eutrophication include the development of planktonic scum and rooted plant biomass, increased algal growth, the death of fish, increased sedimentation, decreased dissolved oxygen concentration, and reductions in water transparency [2].

As the population has increased in coastal watersheds, there has been an increase in the transport of nutrients to lakes, ponds, and estuaries [3,4]. The resulting eutrophication has had many adverse effects within the estuaries [5,6,7]. For example, increased nitrogen loading can lead to phytoplankton blooms [5,7]. These blooms, in turn, lead to the loss of important estuarine habitats, such as sea grass meadows. Sea grass meadow loss is associated with the loss of important commercial shell fish and finfish species, such as cod [8], bay scallops [9], and blue crabs [10]. Eutrophic estuaries can also suffer from anoxia [11], harmful algal blooms, and brown tides [12]. Regime shifts from macrophyte dominance to phytoplankton dominance have also been widely reported, in locations such as Lake Christina, Lake Karibaand and Lake Krankesjon [13,14,15]. These changes are driven by various factors, such as climate, nutrients, lake depth, and lake size [16].

China is a rapidly developing country, and eutrophication has become the most widespread water quality problem [17]. The eutrophication of most lakes and reservoirs in China seriously threatens the regional ecological environment and water security, and has restricted the sustainable development of society and the economy. In past decades, excessive nutrient loading has led to algal blooms in Lake Chao [18,19], Lake Dianchi [20], and Lake Tai [21,22]. The rapidly accelerating pace of eutrophication in lakes across the country has forced the government to set ambitious lake restoration goals [23].

In general, phosphorus (P) supply is thought to regulate algal production in freshwater lakes [24,25,26,27]. In contrast, nitrogen (N) supply is the predominant constraint in many estuaries and shallow marine environments [28,29]. Integrating numerical modeling of P and N based on monitoring, assessing, and predicting the state and changes in watershed nutrients is needed to control eutrophication and management [30].

In China, management tools and policies are proposed and implemented at the provincial level [31]. Therefore, management strategies are best considered within a single region. Compliance with water quality indicators has historically been used as the standard threshold for triggering management measures. This approach does not consider the differences in system responses in different regions. Further, few eutrophication control strategies reflect the differences in lake basin characteristics [32]. Many past studies have considered the quality of a specific water body; however, few studies have considered different functional water areas in a region. Further, using traditional water quality criteria alone is insufficient to measure eutrophication levels. Traditional water quality standards are generally based on toxicological tests. Toxic or harmful substance concentrations or thresholds have served as an assessment baseline; however, there is a negative feedback mechanism between these concentrations and the health of aquatic organisms [33]. The lake nutrient standard is a nutrient variable threshold that corresponds with lake ecosystems; this value reflects the most natural conditions (the state before the large-scale interference of human activity) [34], at which N, P, and other nutrients have fewer toxic effects on aquatic organisms.

This background highlights the importance of developing targeted management measures to reduce eutrophication and potentially harmful phytoplankton activity. Different water bodies have different water quality targets, hydrological characteristics, and different surroundings. Different water quality targets should be set based on different water uses, allowing the adoption of targeted management measures.

This study investigated two different water bodies within one region, to assess their different water targets. These water bodies are located in Tianjin Province in north China, and have different water functions and quality targets. This study selected and analyzed their eutrophication indicators, to identify water quality responses to load reduction and other appropriate management measures. The first water body is the Yuqiao Reservoir, a lake that provides drinking water. It has second class water quality requirements under the GB3838 Chinese water quality standard. The second water body is a core area of the Qilihai Reserve, which is one of the National Nature Reserves. It has first class water quality requirements under the GB3838 Chinese water quality standard.

The goal of this study was to develop an effective watershed management method. First, water quality was assessed using the single factor index method, and then reassessed by analyzing changes of TN/TP to determine the limiting factor. The eutrophication statuses of the two water bodies were then compared based on the comprehensive nutritional state index method and geographic information system (GIS) visualization. Finally, nutrient sources were qualitatively analyzed and management measures were proposed. This included exploring different eutrophication treatment and management measures based on specific loads. There were three key study components: (A) an entrophic index system was established; (B) a time series analysis was conducted to reflect eutrophication trends; and (C) recommendations were made based on contrasts between the eutrophication analysis and nutrient source analysis.

## 2. Materials and Methods

### 2.1. Study Area

**Figure 1:**
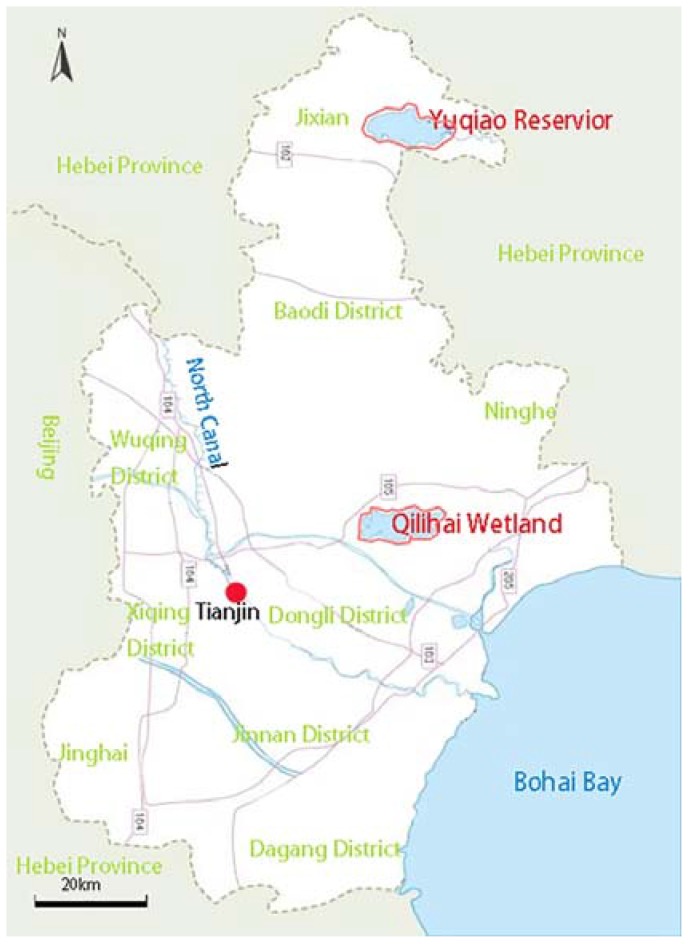
Location of the two studied water bodies located in Tianjin Province in China.

Two water bodies with different functions were selected as the study areas. Figure 1 shows their locations. One is the Yuqiao Reservoir, which provides drinking water and it is located in the north of Tianjin. The other is the Qilihai Wetland, which preserves natural ecological diversity as a national nature protection area. It is located in the middle of Tianjin. The two reservoirs provide different water functions and have separate water quality requirements.

The Yuqiao Reservoir, located in Ji County in China’s Tianjin Province (40°02’ N, 117°25’ E), was formed in 1965 to meet water needs, mainly hydropower and irrigation. It has been the only water supply in Tianjin City (population of more than 10 million) since 1983. The river’s total area is 1627 km^2^. The average water depth is 4.3 m; the total capacity is 1.559 billion m^3^; and the controlled drainage area is approximately 2060 km^2^. The Shahe River, the Lin River and the Li River are the main tributaries. The south bank of the reservoir is steep and the north shore is a relatively flat plain. The reservoir-controlled watershed is a temperate continental monsoon sub-humid climate. The annual average temperature is 10.4 to 11.5 °C; and the annual average precipitation is 748.5 mm, mainly concentrated from June to September [35].

Water safety poses a serious threat in this area. According to the “Regulations on the Protection of Drinking Water Sources” of China, drinking water source protection areas should be classified based on different water quality standards and protection requirements. As a drinking water source protection area, Yuqiao Reservoir is generally divided into primary protected areas and secondary protected areas; if necessary, quasi-protected areas can be added. The regulations note that protected areas at all levels should have clear geographical boundaries, and all levels of protected areas and quasi-protected areas should provide clear water quality standards and deadlines for achieving them. Cross-regional rivers, lakes, reservoirs, water transport channels in the region’s upper reaches should not affect the downstream water quality standards of the Yuqiao Reservoir. Water in the Yuqiao Reservoir is considered a primary protected area. The regulations state that quality standards for primary protected areas should not be lower than those of the “GB3838-88 Surface Water Environmental Quality Standard” Class II standard.

In the protected area of the Yuqiao Reservoir, all activities that may damage or destroy the ecological balance of the environment and forests, water, vegetation and water sources are prohibited. This includes dumping industrial waste, garbage, feces, and other city wastes into the waters; the use of highly toxic and high pesticide residues; the abuse of fertilizers; planting and raising livestock, poultry, and aquaculture activities; and all tourism activities and other activities that may cause water pollution. In addition, no construction may discharge pollutants into the water body; reconstruction projects must reduce pollutant discharges; and original sewage outfalls must cut sewage discharge to ensure that the water quality in the protected area meets prescribed water quality standards.

Wetlands are known as the “kidney of the earth.” Each wetland is a natural community, formed by the interaction of land and water systems [36], and having multiple ecological functions. The Qilihai Wetland (39°17’ N, 117°34’ E) is located in Ninghe County in eastern Tianjin, on the west bank of the Bohai Bay. In 1992, the Qilihai Wetland was approved by the government as a national marine nature reserve. The government is responsible for protecting and managing the natural environment and ecosystems of the Shell embankment, which is an oyster reef containing rare ancient coastal relics and wetlands in China [37]. The Qilihai Wetland covers 45.15 km^2^ and has many ecological functions. It provides water for surrounding rivers, controls flooding, and prevents soil from desertification. The Qilihai Wetland is a national marine area with precious ancient coastal ruins; the wetland’s natural environment and its ecosystem are clear targets for protection and management.

As wetlands have become over-developed by human activities, the biological diversity has decreased, and ecosystem services and other functions have been degenerated [38]. The water environment has been polluted with heavy eutrophication and significant nitrogen, and the water resources have decreased. Based on these factors, the Qilihai Wetland national nature reserve should be managed by the appropriate administrative department within the provincial people’s government or under the State Council [39]. All units and individuals should be obligated to protect nature resources in Qilihai Wetland, and the government should have the right to report and prosecute any unit or individual that has destroyed or occupied the reserve. No production facilities should be built in the core area and buffer zone of the Qilihai Wetland.

As in the reservoir area, in the Qilihai Wetland, it is prohibited to build production facilities that may pollute the environment and destroy resources or landscapes; for other construction projects, the pollutant emissions should not exceed national and local pollutant emission standards. For facilities already built in the study area, any pollutant discharges that exceed state and local government standards should be treated within a prescribed time limit. If damage has been caused, remedial action must be taken. These rules should keep water quality in Qilihai Wetland at or above the first grade of Environmental Quality Standard for Surface Water. The following activities are prohibited in the wetland: logging, grazing, hunting, and fishing, reclamation, burning, quarrying and dredging. In addition, it is prohibited to visit and conduct tourism projects in the Qilihai Wetland. Construction in the peripheral protection zone must not damage the environmental quality of the Qilihai Wetland. If damage has been caused, it must be corrected within a set time limit.

### 2.2. Water Quality of the Two Reservoirs

Many scholars have developed different water quality evaluation methods [40]. Single factor assessment methods are widely used for rivers [41], reservoirs, and lakes. For example, Yang et al. [42] assessed groundwater quality using a single factor and concluded that the groundwater quality in the southern region Ordos basin was generally poor when considered in terms of national groundwater quality standards. A comprehensive index approach, such as the Water Quality Monitor (WQM), was developed by the National Sanitation Foundation (NSF) [43]; other modified, but sound, water quality indicators have been developed based on this approach [44]. Most studies use physical, chemical, and biological characteristics to evaluate existing water quality and pollution statuses [45]. For example, physical characteristics include dissolved oxygen (DO), hydrogen ion concentration (PH), transparency (SD) and temperature [46]. Chemical characteristics include total nitrogen (TP), total phosphorus (TN), chemical oxygen demand (COD), biochemical oxygen demand (BOD), petroleum, and heavy metals [47]. Biological characteristics include chlorophyll a, benthos biomass, and diversity of rare species [48]. TN and TP are often used to indicate the degree of nutrient contamination in a water body; transparency reflects the clarity of the water body; the permanganate index (COD_Mn_) reflects organic and inorganic oxidizable substance pollution in the water; and chlorophyll a (Chl-a) reflects the amount of phytoplankton in the water. We have used these as indicators to assess the water quality of the two reservoirs.

**Figure 2:**
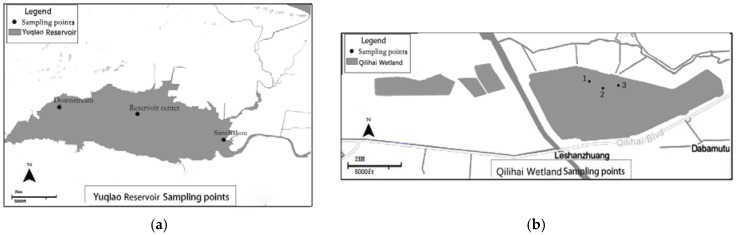
(**a**) Sampling points for Yuqiao Reservoir; (**b**) Sampling points for Qilihai Wetland Nature Reserve.

Based on the hydraulic characteristics of the surface water, three monitoring points (Figure 2a) were established in the Yuqiao Reservoir. We collected data from the three monitoring points in the Sanchakou, the reservoir center, and the dam. The average measures across the three locations provided the data for analysis. In addition, we set up three monitoring points around Bird Island (Figure 2b) in Qilihai Wetland Nature Reserve and used the annual average of the three monitoring points to reflect water quality.

The single factor index method is a relatively simple and useful method to assess water quality [49] and is used to evaluate the water quality in this study.

The single factor index evaluation method is presented as:

$$S_{i,j}=\frac{C_{i,j}}{C_{s,i}}$$

In this expression, *S_i,j_* is the standard index of the water quality parameter *i* at point *j*. *C_i,j_* is the concentration of water quality parameter *i* at *j* point (mg/L); *C_s,i_* is water quality standard *i*, (mg/L), whose value is regulated by the standard of “surface water environmental quality” (GB3838-2002) in Table 1.

### 2.3. Eutrophication Situation

Different scholars have proposed many eutrophication evaluation models [50,51,52,53]. Yang et al. used a series of artistic neural networks to develop an eutrophication assessment model for aquaculture water areas [54]. Wu et al. established a hybrid model combining water quality indicators and ecological response indicators to assess eutrophication, and then applied it to assess the status of eutrophication from 2007 to 2008 in the southwest Bohai Sea [55]. Huo et al. established a region-specific lake eutrophication assessment standard using a frequency distribution method based on Chl-a concentration [56]. Liu et al. used a water quality modeling-based scenario analysis approach to quantitatively evaluate how eutrophication in Lake Dianchi responded to an under-construction water diversion project [57]. These evaluation methods have advantages and disadvantages; many are subjective, and calculations are complex, error-prone, and inconvenient.

Based on the China Environmental Monitoring Center’s “Lake Reservoir eutrophication evaluation methods and classification of technical requirements,” many scholars use an integrated nutritional status index method to assess eutrophication in lakes. The comprehensive nutritional status index method has matured and the evaluation range is more comprehensive than other nutritional index methods. The evaluation factors comprehensively consider indexes such as TN, TP, SD, Chl-a and COD_MN_. Further, the method has mitigated the single evaluation factor’s lack of evaluation. We used the eutrophication index to assess the eutrophication of the two reservoirs. The equation is:

$$\mathrm{TLI}\;(\sum )=\sum_{j=1}^{m}\;W_{j}\times \mathrm{TLI}(j)$$

In this expression, TLI (Σ) is the comprehensive nutritional status index; W_j_ is a normalized weighted value of index j; and TLI (j) is the eutrophication evaluation universal index of index j.

Studies by Jin summarize the correlation coefficient between the Chl-a and other parameters in China’s lakes and reservoirs; these provide an important basis for calculating weights [58]. With Chl-a as the reference parameter, the normalized correlation weight of the index j parameter is calculated as follows:

$$W_{j}=\frac{r^{2}\mathrm{ij}}{\sum_{j=1}^{m}r^{2}\;\mathrm{ij}}$$

In this expression, r_ij_ is the correlation coefficient between the index j parameter and the reference parameter Chl-a; m is the number of evaluation parameters. Table 2 shows the correlation between the Chl-a and other parameters in Chinese lakes (reservoirs).

After calculating the eutrophication index, we classified reservoir eutrophication into five different grades according to the grading standards. Table 3 lists these grades. In the same nutritional state, the higher the index value, the more severe the nutritional level.

## 3. Results and Discussion

### 3.1. Physical and Chemical Characteristics of Water Quality

**Figure 3:**
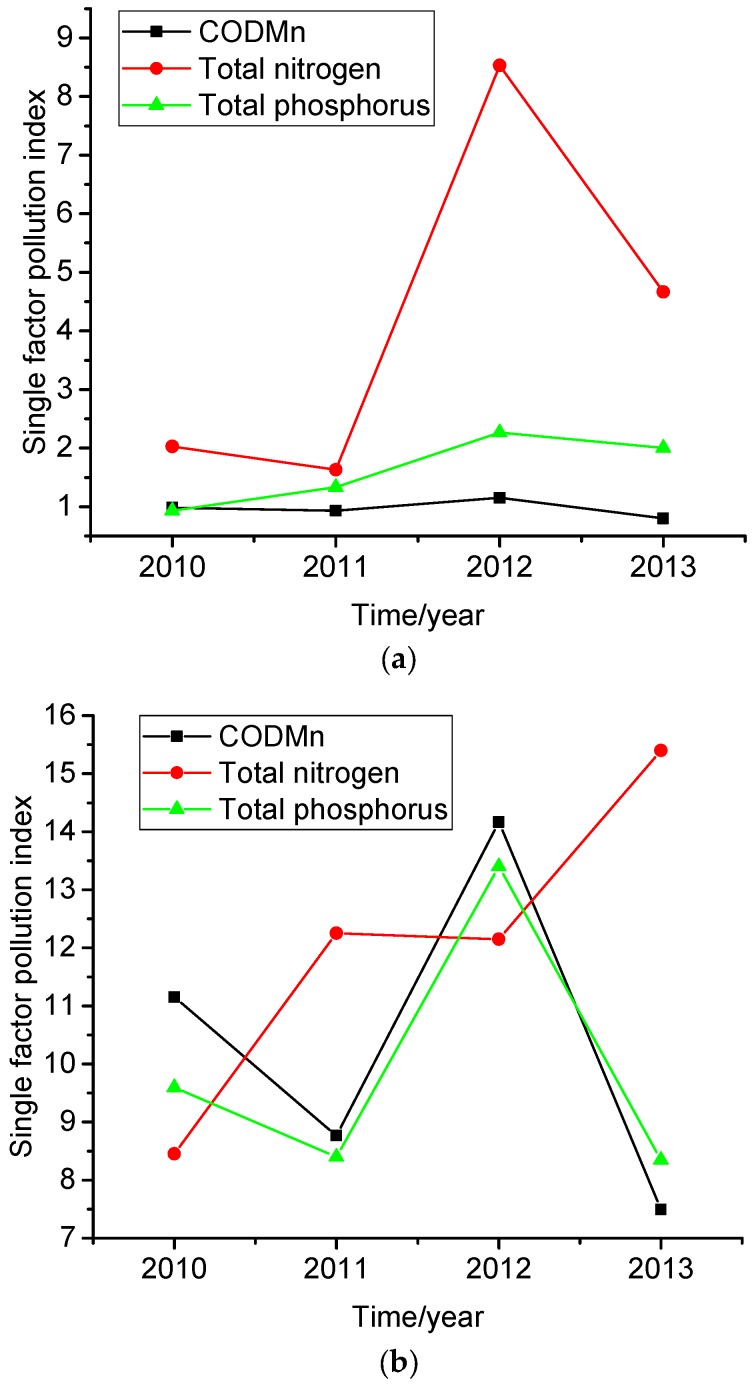
(**a**) Trend in the single factor pollution index for Yuqiao Reservoir; (**b**) Trend in the single factor pollution index for Qilihai Wetland Nature Reserve.

Table 4 and Table 5 show the physicochemical characteristics of the water samples; Figure 3a,b show the Single Factor Pollution Index. According to the surface water environmental quality standard classification and water environment basic project standard limit (National Standard of the People’s Republic of China, Environmental Quality Standards for Surface Water, GB3838-2002), the Yuqiao Reservoir’s surface water quality is consistent with second class water environmental quality standards. For the Qilihai Wetland, the surface water quality is consistent with first class water environmental quality standards. 

The average concentration of COD_Mn_ in the Yuqiao Reservoir ranged from 3.2 to 4.6 mg/L; the single factor pollution index of COD_Mn_ ranged from 0.800 to 1.151 with an average value of 0.967. In 2012, the average concentration of COD_Mn_ exceeded the limit for water quality criteria Grade II, but in 2013 there was a significant decline. The COD_Mn_ pollution index at the sampling stations met the expected class of water quality standards except in 2012. The TN concentration was higher than the limit for water quality criteria Grade II during the monitoring period. The single factor pollution index of TN ranged from 1.633 to 8.533, with an average value of 4.215. The single factor pollution index exceeded Grade II of the water quality standards for four years. This indicates the significance of nitrogen pollution.

In recent years, the TP concentration continued to rise. The single factor pollution index of TP ranged from 0.933 to 2.267 with an average value of 1.633. From 2011 to 2013, the single factor pollution index exceeded the Grade II of water quality standards. Further, the transparency value was lower than the lake eutrophication standard of 2.4 m, indicating that the reservoir was eutrophic. Table 4 and Figure 3b show the COD_Mn,_ TN and TP concentration trends in the Qilihai Wetland Nature Reserve; the concentrations fluctuate, but all of them exceed the surface water environmental quality standards of the first Grade and some exceed Grade IV. In 2011 to 2013, the nitrogen concentration significantly exceeded Grade V of the surface water environmental quality standard.

The single factor pollution index of COD_Mn_ ranged from 7.495 to 14.165 with an average value of 10.390. The single factor pollution index of TN ranged from 8.45 to 15.40 with an average value of 12.06. The single factor pollution index of TP ranged from 8.35 to 13.40, with an average value of 9.91. And the transparency was also lower than the lake eutrophication standard which is 2.4 m. Of these values, the nitrogen concentration is particularly significant, indicating the serious pollution impacting the water quality of Qilihai Wetland. TN and TP were the main factors in these two water areas that exceed the standard.

As mentioned above, the single factor indexes of COD_Mn_ and TP in Qilihai Wetland were almost 10 times the indexes of Yuqiao Reservoir. There are many reasons for high COD_Mn_ concentrations in Qilihai Wetland, including industrial and agricultural activities, other discharges, domestic sewage, and rubbish. In addition, the chlorophyll concentration in Qilihai Wetland is much higher than the concentration in Yuqiao Reservoir. This may be because the industrial wastewater and domestic sewage surrounding Qilihai Wetland and the farmland shore generated more surface runoff with more nutrients. This supports algae growth and reproduction. The results of the single factor pollution index method follow in Figure 3.

### 3.2. Ratio Changes of TN/TP

The TN/TP ratio continuously fluctuated in the two water bodies. The total nitrogen levels were related to ammonia nitrogen and nitrite levels in water. Ammonia nitrogen in the water was also not stable. After biological assimilation, the remaining dissolved oxygen was present in sufficient conditions. Many nitrifying bacteria oxidized to nitrite nitrogen. In the flood season, a variety of nitrifying bacteria and dissolved oxygen were reduced, leading to ammonia nitrogen and nitrite nitrogen conversion. The transformation process resulted in excessively large ammonia and nitrite nitrogen concentrations over the course of a few months. Therefore, the total nitrogen increased; on the contrary, it decreased. However, during the drought period, due to the role of denitrification, anaerobic, or hypoxia, nitrate nitrogen (NO_3_^−^) served as an acceptor. This reduced other gaseous oxides of nitrogen or nitrogen. This leads to the reduction of nitrate nitrogen and total nitrogen in water, gradually reducing the water storage capacity. The reservoir’s water storage capacity affects TP changes, because when the water storage capacity is gradually reduced, it promotes sediment redox, mineral dissolution and adsorption, and bacteria and microbe metabolism. This includes hydrodynamics or bioturbation [59]. This resulted in phosphorus being released from the sediments; the phosphorous then becomes an endogenous source contributing to the water phosphorus load.

Many factors modify the demand and supply of N and P for phytoplankton in the upper lake waters. However, the TN/TP ratio helps predict which of the two most commonly limiting macronutrients will become the most growth limiting under well-illuminated, stratified conditions [60]. In addition, the TN/TP ratio is an important factor affecting algal growth; it reflects the production cycle and phytoplankton production in the water body [61]. Researchers generally believe that when the TN/TP ratio is 10:1–25:1, there is a linear correlation between algae growth and nitrogen and phosphorus concentration. This benefits algae growth, which is prone to eutrophication [62].

During the monitoring period, the TN/TP ratios in the Yuqiao Reservoir progressed from 43, 25, 75, and 47 from 2010 to 2013. The TN/TP ratios in the Qilihai Wetland progressed from 9, 15, 9, and 18 from 2010 to 2013. The TN/TP ratios in Qilihai Wetland ranged 10:1 and 25:1 from 2010 to 2013, favoring algae growth. The TN/TP ratio in the Yuqiao Reservoir did not support algae growth during the monitoring period. Algae growth and reproduction processes require a variety of nutritional elements. If a certain nutrient is relatively low, growth and reproduction processes will be limited. This element is called the “main limiting nutrient” [60]. Laguna et al. [63] noted that when TN/TP exceeds 16, the phosphorus is in a restricted state; when TN/TP is less than 16, the nitrogen is in a restricted state. Thus, phosphorus was the limiting factor in the Yuqiao Reservoir; nitrogen was the limiting factor in the Qilihai Wetland.

### 3.3. Eutrophication Evaluation

Table 6 shows the TLI of Yuqiao Reservoir from 2010 to 2013. TN, Chl-a, and SD level (the three nutritional status indexes) were high. The degree of eutrophication in the Yuqiao Reservoir was highest in 2012, with levels decreasing in decreasing degrees in 2013, 2010, and 2011. Water quality was in a mesotrophic state (30 ≤ TLI (∑) ≤ 50) except during 2012 and 2013. The water quality in 2012 and 2013 was in light eutrophic state (50 < TLI (∑) ≤ 60). This shows that from 2010 to 2013, Yuqiao Reservoir water quality ranged between a mesotrophic and light eutrophic state. Eutrophication in 2012 was the most serious. This may have been because of the self-purification capacity [64] of the Yuqiao Reservoir, leading to an overall declining trend for the reservoir. Overall, the Yuqiao Reservoir water quality in 2010 to 2013 was still relatively good.

Table 7 shows the TLI of the Qilihai Wetland Nature Reserve from 2010 to 2013. The nutritional status index of TN showed a significant upward trend; strict measures are recommended to regain and maintain control. The nutritional status index of TP, COD_Mn_, Chl-a, and SD fluctuated frequently across the four years, indicating an unstable water environment. The degree of eutrophication of Qilihai Wetland was highest in 2013, and declined across 2011, 2012, and 2010. The wetland was in a hyper eutrophic state (TLI (∑) > 70) for a long time, and the eutrophication trend continues to rise. The nutritional indexes all significantly exceeded the standard. This shows that from 2010 to 2013, the water quality of Qilihai Wetland Reserve was significantly polluted, and already in a hyper eutrophic state.

**Figure 4:**
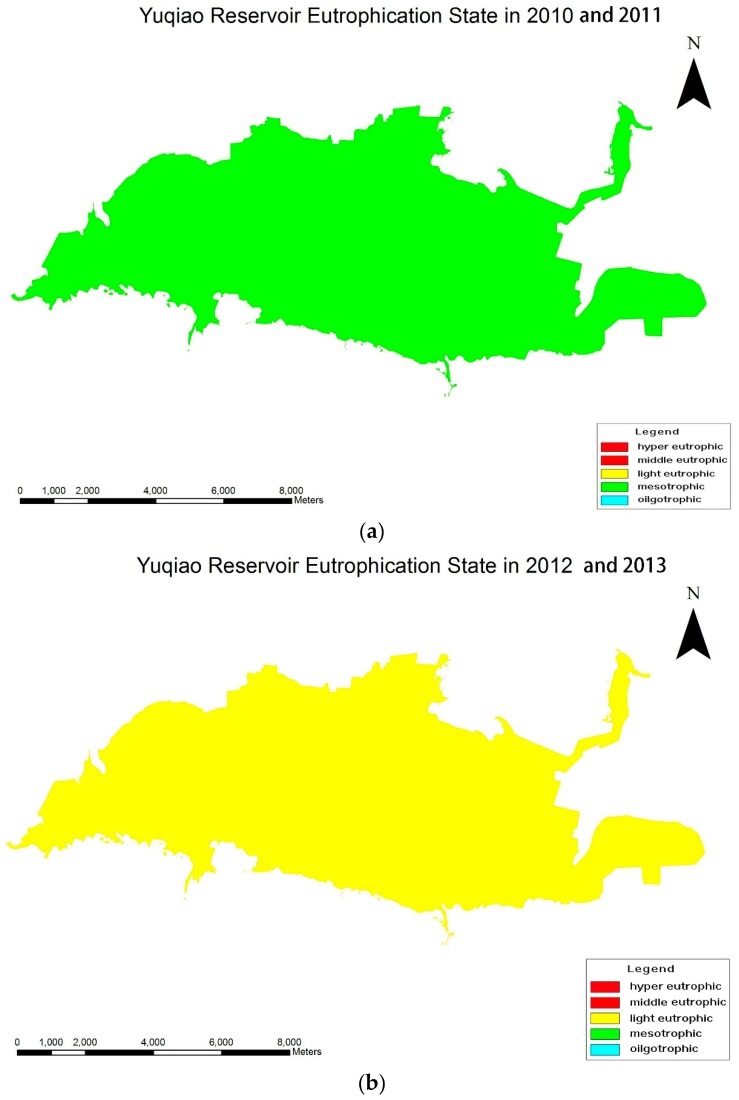

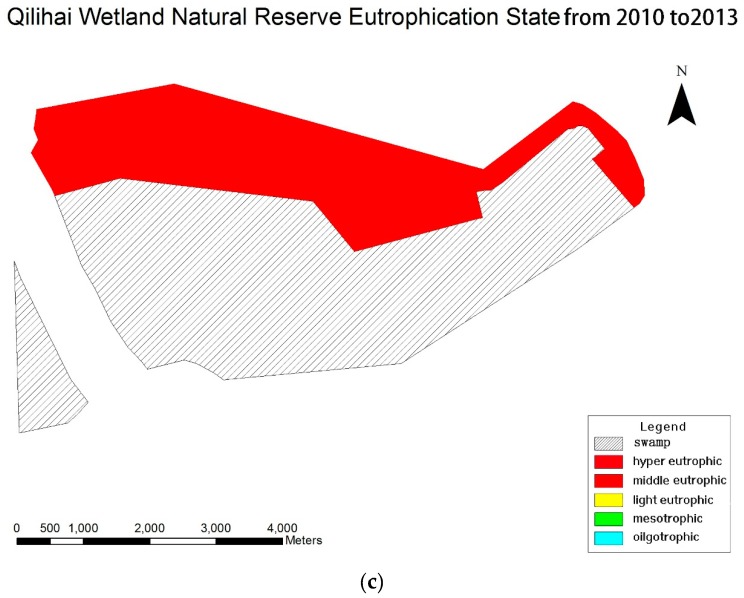
(**a**) Eutrophication trends in Yuqiao Reservoir in 2010 and 2011; (**b**) Eutrophication trends in Yuqiao Reservoir in 2012 and 2013; (**c**) Eutrophication trends in Qilihai Wetland Nature Reserve from 2010 to 2013.

According to Environmental Quality Standards for Surface Water (GB3838-2002), Qilihai Wetland Nature Reserve falls within the scope of the first class. Yuqiao Reservoir falls within the second or third class. With respect to monitoring results, the degree of eutrophication of Qilihai Wetland Nature Reserve was far above the Yuqiao Reservoir (Figure 4).

### 3.4. Analysis of Nutrient Sources

The information above indicates that nutrients are the main cause of eutrophication. However, the nutrient inputs differ between different water areas. As such, we analyzed nutrient sources from both internal and external sources.

#### 3.4.1. Internal Sources

Sediment is an internal source of eutrophication, both in the Qilihai Wetland and in the Yuqiao Reservoir. Sediments contain abundant nutrients and accumulate year to year. Accumulation is particularly high in the summer, when there is more light, the water temperature is high, and nutrients are easily released [65]. Therefore, the nutrients in the sediments are a driver for eutrophication.

Cheng [66] determined the total amount of contaminated sediment in Yuqiao Reservoir, which contributes less to the water pollution than other sources. Past studies have shown that the total nitrogen in the Qilihai wetland sediments was relatively enriched [67]. However, the nitrogen analysis presented above is the limiting factor in the Qilihai Wetland, which is a relatively low nutrient level relative to algae growth. Therefore, the sediment pollution contributes less to the nutrient source. Nitrogen accumulation in the Qilihai Wetland is also due to human activities [68]. The sediment contributes little to the nutrient sources of the two water areas; the main source comes from external sources.

#### 3.4.2. External Sources

Point source controls around the Qilihai Wetland and the Yuqiao Reservoir have achieved certain results, gradually reducing industrial point source pollution around the Qilihai Wetland and Yuqiao Reservoir [35]. Non-point source pollution in the region comes from different sources, the pollution load is large, and the pollution rate is very high.

In the Yuqiao Reservoir, where water inflow is generally stable, all nutrient types increased. There are 129 villages close to the reservoir’s water environment [69]. As the number of urban residents has increased, the amount of phosphorus containing detergents and domestic sewage discharge has also increased, but the sewage treatment rate remained at almost zero. As the economy developed, there was an increase in the cultivated land upstream of the Yuqiao Reservoir, with extended planting times [70]. This increased the use of different chemical fertilizers and pesticide varieties; as the dosage increased, farmland fertilizer was lost through surface runoff into the Yuqiao Reservoir. More recently, the rapid development of aquaculture has led to the discharge of fish bait, small amounts of fertilizer, and other fish products into the reservoir. This has accelerated the eutrophication rate. In addition, there has been a decrease in the area of the Yuqiao Reservoir covered by aquatic plants [69], narrowing the aquatic plant growth range. One plant species dominates the area, providing a basis for the further algae growth.

The Qilihai Wetland Nature Reserve is one of the most eco-diverse ecological landscapes in Tianjin. As such, it attracts tourists from all over the world, and tourism has been rapidly developing [71]. However, excessive tourism and tourists transfer the metabolites of tourist activities into the Qilhai Wetland system, destroying the water environment. Tourism development has also expanded the number and variety of dining establishments around the wetland. The wastes produced by dining establishments further pollute the Qilihai Wetland’s water quality. Because of rapid aquaculture development, there is also fishpond pollution in the Qilihai Wetland Nature Reserve [72]. The fish bait and other fertilizer used to feed fish hinder water resources flow. This reduces the water’s self-purification capacity, giving rise to water contamination. Similarly, the poor management of aquatic organism distribution has also increased eutrophication of the Qilihai Wetland.

### 3.5. Management and Measures

Because it is the only water source in Tianjin, the water quality in the Yuqiao Reservoir is closely linked to the life and health of its residents. This has attracted the attention of both government agencies and residents, who work together to protect the Yuqiao Reservoir water quality. Several industries around the reservoir that significantly polluted the water have been dismantled [66]. In addition, residents work to consciously enhance reservoir awareness.

There are many laws and regulations governing nature reserves, however, there are not enough management institutions in the Qilihai Wetland to fully implement relevant conservation policies. Due to a decentralized protection approach, the lack of sufficient infrastructure, and a lack of strong financial support, the water pollution has become more serious. In addition, outreach about protected areas has been insufficient; residents lack a sense of protection consciousness [73,74]. These factors have resulted in greater pollution in the Qilihai Wetland than in the Yuqiao Reservoir. To prevent the deterioration of eutrophication in Yuqiao Reservoir and in the Qilihai Wetland natural reserve, stricter treatment methods and management measures are needed.

Appropriate control and management measures are needed; however, it is unwise to control either nitrogen or phosphorus pollution alone. Based on the situation around the two water areas, management policies are needed to improve water quality.

Appropriate measures are needed to introduce controls for different pollution sources. For industrial pollution, the government should allocate special funds to address the pollution, close polluting enterprises, and use advanced technology to reduce pollution emissions and production. With respect to urban waste, the government should unify garbage collection and establish sites for sanitary landfills. To address source pollution, the government can remediate water quality through ecological restoration, dredging, and other technical means [75]. We recommend rehabilitating water quality based on wetland type and water characteristics [76]. This may include establishing relevant laws and regulations to regulate aquaculture and fisheries, strengthening poultry manure management, applying the rational use of fertilizers and pesticides, strengthening the ability of vegetation to absorb excess nutrients, paying attention to the rational distribution of aquatic organisms, and controlling soil erosion.

In addition to these steps, the government should strengthen governance and management, and at the same time, strengthen awareness. This includes encouraging local people to learn more about environmental protection. Further, increasing the application of remote sensing, GIS and other technical means will assist in eutrophication management, improve the targeted effects of management, and promote effective management. In addition, due to the location and other characteristics of the Yuqiao Reservoir, it is important to pay attention to and control nitrogen and phosphorus pollution in the upstream river and around the river. It is also important to prohibit the development of unsustainable tourism and to plan wetland structures rationally in the Qilihai Wetland.

Wetland water quality parameters are often related to hydrological processes and the growth season of wetland vegetation [74]. Effective management requires carrying out dynamic monitoring of the Qilihai Wetland water quality, establishing automatic prediction and alarm systems, and strengthening research. Managing water functions is an important way to manage water resources. A hierarchical management system combines river basin management and regional management to form a water resources management system. Therefore, for the Qilihai Wetland Nature Reserve, which has higher water quality requirements, management should be strengthened. At the same time, the government cannot ignore the Yuqiao Reservoir management. To improve water quality in the long term and bring water quality in line with water quality standards, governments and stakeholders must restore and protect environmental quality through management tools, scientific research, and technology projects. The government should also establish a protection target responsibility system and quantitative assessment management approach. For different functional water areas in a region, the government should be hierarchically managed and water areas should be managed to achieve higher water quality standards.

This study does have a few limitations. First, the water quality assessment indicators could be increased, allowing a more comprehensive analysis of results. In addition, while this article proposes unilateral solutions, a more detailed quantitative or qualitative analysis would better determine pollution mechanisms and more specific solutions. As the self-purification capacity of the Yuqiao Reservoir and Qilihai Wetland Nature Reserve decline, further study of the ecological evaluation index could accurately describe the problems. This highlights additional areas for future study.

## 4. Conclusions

This study analyzed two typical water bodies in Tianjin, China, with different water quality targets. Water quality was first assessed using the single factor index method; changes in TN/TP were then analyzed to determine the limiting factor. Next, the eutrophication status of the two water areas were compared, using a comprehensive nutritional state index method and geographic information system (GIS) visualization. Finally, nutrient sources were qualitatively analyzed and management measures were proposed.

In the Yuqiao Reservoir, the COD_Mn_ concentration met the water quality standard every year except 2012. The TN concentration fluctuated frequently; it consistently exceeded the standard. The overall TP concentration exceeded the standard from 2011 to 2013. The Chl-a concentration was consistent with the regular pattern .In contrast, the COD_Mn_, TN, and TP concentrations in the Qilihai Wetland Nature Reserve fluctuated, but they all exceeded the surface water environmental quality standards of the first level, as well as standards at Grade III or Grade IV. The change of Chl-a concentration was particularly problematic. The water quality in the Qilihai Wetland was particularly serious. The single factor index indicates that TN and TP were the main factors in the two areas that led to the standard being exceeded.

The TN/TP ratio in the water bodies in the two study areas fluctuated continuously. During the monitoring period, the TN/TP ratios in the Yuqiao Reservoir from 2010 to 2013 were 43, 25, 75, and 47 respectively. The TN/TP ratios in the Qilihai Wetland from 2010 to 2013 were 9, 15, 9, and 18 respectively. Thus, phosphorus was the limiting factor in the Yuqiao Reservoir; nitrogen was the limiting factor in the Qilihai Wetland.

From 2010 to 2011, the eutrophication level of the Yuqiao Reservoir was consistently in the mesotrophic state. In 2012 and 2013, it was in a light eutrophic state. Because the surface water environmental quality standard was classified as the first class, the Qilihai Wetland Nature Reserve was consistently in a hyper eutrophic state from 2010 to 2013. Although there were some eutrophication problems in the Yuqiao Reservoir, the pollution in the Qilihai Wetland was more serious.

The analysis shows that external pollution is the main source of pollution in the Yuqiao Reservoir and the Qilihai Wetland. The main nutrient sources for the Yuqiao Reservoir come from the upper reaches of the rivers that feed it; the nutrient load is excessive due to aquaculture development and the poorly managed distribution of aquatic organisms. In the Qilihai Wetland, nutrients are mainly caused by excessive tourism, aquaculture, and agriculture development.

Targeted management measures are needed to prevent the water quality of the Yuqiao Reservoir and the Qilihai Wetland from continuing to deteriorate. These measures should be driven based on different pollution sources; they require, however, an improvement in the area’s hierarchical management system.

Water quality needs to meet the requisite standards and prevent harm to the human body. This involves protecting water quality, and maintaining the economic and ecological benefits of wetlands and reservoirs. To maintain both ecological and socio-economic significance, it is important to learn and master the status quo and improve management efficiencies before implementing local government policies. This is the primary message from this study.

## Figures and Tables

**Table 1:** *C_s,j_* of the two reservoirs (mg/L).

Indicators	COD_Mn_	TN	TP	Chl-a	SD
Grade I	2	0.2	0.02	-	-
Grade II	4	0.5	0.025	-	-
Grade III	6	1	0.05	-	-

**Table 2:** The correlation between lake parameters (reservoir), Chl-a, and r_ij_, r^2^_ij_ and weights (W_j_).

Parameter	Chla	TP	TN	SD	COD_Mn_
r_ij_	1	0.84	0.82	−0.83	0.83
r^2^_ij_	1	0.7056	0.6724	0.6889	0.6889
W_j_	0.26626	0.18787	0.17903	0.18342	0.18342

**Table 3:** Grading standards of eutrophication.

Grades	Values
Oligotrophic	TLI (∑) < 30
Mesotrophic	30 ≤ TLI (∑) ≤ 50
light eutrophic	50 < TLI (∑) ≤ 60
middle eutrophic	60 < TLI (∑) ≤ 70
hyper eutrophic	TLI (op) > 70

**Table 4:** Monitoring results of the water environment single indices in Yuqiao Reservoir.

Test Items	2010	2011	2012	2013	Standard Interval
**COD_Mn_**	3.933333	3.733333	4.603333	3.2	II ≤ 4 III ≤ 6
**TN**	1.013667	0.816667	4.266667	2.333333	II ≤ 0.5 III ≤ 1
**TP**	0.023333	0.033333	0.056667	0.05	II ≤ 0.025 III ≤ 0.05
**Chl-a**	13.53333	10.41	16.4	10.66667	-
**SD**	0.5	0.8	0.83	0.9	-

**Table 5:** Monitoring results of the water environment single indices in Qilihai Wetland Nature Reserve.

Test Items	2010	2011	2012	2013	Standard Interval
**COD_Mn_**	22.30	17.53	28.33	14.99	I ≤ 2 V ≤ 15
**TN**	1.69	2.45	2.43	3.08	I ≤ 0.2 1.5 < V ≤ 2.0
**TP**	0.192	0.168	0.268	0.167	I ≤ 0.02 0.2 < IV ≤ 0.3
**Chl-a**	87.209	33.45	92.85	54.70	—
**SD**	0.38	0.18	1.23	0.27	—

**Table 6:** Nutrient state index of Yuqiao Reservoir from 2010 to 2013.

Year	TN	TP	COD_Mn_	Chl-a	SD	TLI (∑)
**2010**	54.75995	33.33215	37.53206	53.29199	64.62706	48.99333
**2011**	51.09924	39.12455	36.14339	50.44245	55.50898	46.74031
**2012**	79.10711	47.74196	41.71763	55.37848	54.79479	55.57921
**2013**	68.88323	45.70931	32.04144	50.70696	53.22399	50.06019

**Table 7:** Nutrient state index of Qilihai Wetland Nature Reserve from 2010 to 2013.

Year	TN	TP	COD_Mn_	Chl-a	SD	TLI (∑)
**2010**	63.4189	67.55978	83.70305	73.52582	69.95113	70.80658
**2011**	69.70973	65.39123	77.29874	63.11916	84.44709	71.23871
**2012**	69.57088	72.97568	90.07175	74.2065	47.16393	71.09521
**2013**	73.58631	65.29427	77.23796	68.46024	76.58107	71.8827
